# Influence of spousal educational disparities on intimate partner violence (IPV) against pregnant women: a study of 30 countries

**DOI:** 10.1038/s41598-024-84867-2

**Published:** 2025-01-15

**Authors:** M. D. Nahid Hassan Nishan, M. Z. E. M. Naser Uddin Ahmed, Saidur Rahman Mashreky, Koustuv Dalal

**Affiliations:** 1 Department of Public Health, North South University, Dhaka, Bangladesh; 2 Division of Public Health Science, Department of Health Sciences, Mid Sweden University, Sundsvall, Sweden; 3 University of Johannesburg, Johannesburg, South Africa

**Keywords:** Educational disparities, Intimate partner violence, LMIC, Spousal education, Violence against pregnant women, Risk factors, Epidemiology

## Abstract

Intimate Partner Violence (IPV) during pregnancy poses a serious threat to maternal health, particularly in low- and lower-middle-income countries (LMICs). Despite these known risks, the role of spousal educational differences in IPV during pregnancy remains poorly understood. This study aimed to examine this influence, analyzing data from multiple countries across five continents. This study utilized data from Demographic and Health Surveys (DHS) focusing on lower and LMIC countries. DHS employs two-stage sampling to gather comprehensive health data. Thirty countries from five regions were selected. Covariates like husband’s age, residence, wealth, education, husband’s working status, husband’s education, and spousal educational gap were considered. Cross-sectional survey design was considered. Chi-square test was done to find the association between IPV and covariates. Binary logistic regression was used to assess whether the independent variable is related to spousal educational disparity and other covariates of IPV during pregnancy. Out of 152,643 (weighted) pregnant women from all five continents, 8357 (weighted) experienced IPV during pregnancy. IPV is most prevalent in Papua New Guinea (17.01%; 95% CI 15.76–18.38%)), while least prevalent in Cambodia (0.99%; 95% CI 0.88–1.10%)). Overall, the IPV prevalence was (5.47%; 95% CI 5.30–5.65%)). Educational disparity and socioeconomic factors play a significant role in encountering IPV during pregnancy. This study revealed complex, region-specific effects on violence likelihood, emphasizing implications for policymakers and practitioners addressing IPV. Education disparity emerged as a significant factor; lower-educated couples exhibit increased abusive behavior.

## Introduction

Intimate Partner Violence (IPV) is a significant widespread issue, making an alarming global public health concern, and affecting countless women worldwide^1^. IPV is increasing daily and gaining recognition as a serious threat with significant societal and clinical implications^1^. It encountered various forms of abuse, including physical, sexual, emotional, and economic mistreatment, inflicted by a current or former partner. The impact of IPV can have serious consequences for the health and welfare of women and their children, especially during pregnancy^2^. It can result in several complications like perinatal depression, anxiety, post-traumatic stress disorder (PTSD), and adverse birth outcomes^3^. Women who experience IPV during pregnancy are three times more likely to exhibit those symptoms compared to those who do not^4^. Studies indicate that women subjected to abuse during pregnancy are at a significantly higher risk of delayed or insufficient prenatal care. Women who experience IPV during pregnancy are twice as likely to either postpone the initiation of prenatal care or miss appointments entirely, often not seeking care until the third trimester^4–6^. Additionally, nearly half of the women facing IPV (45%) miss three or more prenatal visits, compared to (28%) of no-abused women, highlighting the severe impact of IPV on maternal health^4^.

Globally, IPV does not discriminate; it affects women across all demographics regardless of age, race, or socioeconomic status^7–11^. Recent studies further highlight that IPV prevalence varies widely across regions, with Sub-Saharan Africa and South Asia reporting some of the highest rates due to entrenched cultural norms and limited access to resources^12,13^. This regional variation underscores the need for a contextualized understanding of IPV risk factors. However, the risks are often heightened in low and lower-middle-income countries (LMICs), where gender inequality, poverty, and traditional cultural norms may exacerbate the problem^14^. A study revealed that in lower-middle-income countries like Bangladesh, 42% of male adolescents justified wife beating, while in India, 51% supported such behavior^15^. In Nepal, 28% of respondents also condoned wife beating, often for reasons such as the wife leaving the house without permission or refusing to have sex^15^. These attitudes are rooted in deeply entrenched patriarchal systems that normalize male dominance and reinforce the subjugation of women. Such gender imbalances create environments where women lack agency and protection, perpetuating cycles of abuse and marginalization. This societal acceptance of violence intensifies the vulnerability of women, particularly during pregnancy.

Men have allocations to resources and decision-making power in the family^16^. A theory called the “feminist theory” claims that IPV may be caused by an unbalanced allocation of gender power, which can be visible in a patriarchal society where it is assumed that a man abuses his wife to gain power and control^16^. Despite the well-documented risks of IPV during pregnancy, there remains a lack of understanding about whether spousal educational disparities influence the likelihood of such violence. Educational disparities can create power imbalances in relationships, which are often associated with an increased risk of IPV. Studies have shown that unequal educational levels between partners may lead to conflict, with higher education among women challenging traditional gender roles, potentially increasing IPV risk in patriarchal societies^15,17^. Conversely, higher education in both partners is often linked to lower IPV risk, as it promotes equitable decision-making and respect for gender equality^18^. These dynamics make spousal educational disparities a critical area of study, particularly in LMICs, and there has been little exploration into how differing educational levels between partners may contribute to IPV, particularly during pregnancy—a time when women are most vulnerable. This gap in the literature forms the central focus of this study.

Therefore, this study aims to fill this gap by evaluating the role of spousal educational disparities in influencing IPV during pregnancy. This study offers a comprehensive global perspective by analyzing data from 30 countries across five distinct regions—Sub-Saharan Africa, South/Southeast Asia, Latin America and the Caribbean, North Africa/West Asia, and Oceania. Importantly, we focus on low and LMICs, where economic and cultural factors intersect to create complex dynamics around gender, power, and education^19^.

Analyzing datasets derived from low and LMIC countries between 2011 and 2023, using the Demographics and Health Survey (DHS) will enhance our understanding of the covariates associated with IPV during pregnancy and the role of spousal educational backgrounds in this form of abuse. It is believed that this study is considered highly important as it provides valuable insights into global patterns and offers practical recommendations for policymakers and practitioners. It also presents a unique perspective with significant implications for public health at both national and international levels.

## Methodology

### Study description

This study utilized secondary datasets from the DHS. The classification of low-income and LMICs was based on the World Bank’s criteria^20^ identifying 61 countries of interest initially from the DHS. Upon further analysis of each country’s database, 30 countries were selected for inclusion as they provided all the relevant data necessary for this study’s primary focus. The regional country lists were aligned with the World Bank’s classifications.

Countries included in the study from DHS databases:

Sub-Sahara African Region: Angola 2015-16^21^, Benin 2017-18^22^, Burundi 2016-17^23^, Cameroon 2018^24^, Comoros 2012^25^, Cote d’Ivoire 2011-12^26^, Ethiopia 2016^27^, Kenya 2022^28^, Madagascar 2021^29^, Malawi 2015-16^30^, Mali 2018^31^, Mauritania 2019-21^32^, Mozambique 2011^33^, Nigeria 2018^34^, Sierra Leone 2019^35^, Tanzania 2015-16^36^, Togo 2013-14^37^, Uganda 2016^38^, Zambia 2018^39^, Zimbabwe 2015^40^.

South and Southeast Asian Region: Cambodia 2021-22^41^, Nepal 2022^42^, Pakistan 2017-18^43^, Philippines 2022^44^, Timor-Leste 2016^45^.

Latin America & Caribbean Region: Haiti 2016-17^46^, Honduras 2011-12^47^.

North Africa/West Asia Region: Egypt 2014^48^, Jordan 2017-18^49^, Oceania: Papua New Guinea 2016-18^50^.

### Study design

This study adopts a cross-sectional design. The DHS surveys are globally recognized for collecting comprehensive and nationally representative data on various health indicators in developing countries. The data collection process in the DHS surveys follows a two-stage sampling design based on enumeration areas (EAs). The information systems of the DHS are intentionally overseen according to the parameters set by the EA framework. This framework guarantees that these systems are in sync with the mission, goals, and objectives of DHS while prioritizing safeguarding individual personal information (PII) to ensure privacy protection. DHS provides training to handle identifiable information, privacy regulation, and rights and remedies regarding privacy policy. The DHS obtains PII from individuals through direct interaction or communication, called data collection - interview.

### Study participant

DHS gathers data from different health domains, including records for households, Children, men, and women. This study specifically focused on pregnant women who had experienced IPV during their pregnancy, as identified in the individual (IR) datasets. To acquire comprehensive results, all data were recoded and cleaned, any missing values were removed, and only the variables needed for the analysis were kept. A total of 152,643 (weighted) participants were sampled for our analysis.

### Variable of interest

The primary outcome variable in this study was the experience of IPV during pregnancy, derived from the DHS dataset. The analysis included women aged 15–49 who were selected and interviewed as part of the IPV module. The women who had ever been pregnant were identified based on whether they had given birth, were currently pregnant, or had experienced a terminated pregnancy, and only those who reported experiencing physical IPV during their pregnancy were included in the analysis.

To assess IPV during pregnancy, respondents were classified based on whether they had been physically hurt by their husbands during any pregnancy. The outcome variable was recoded into a binary format: 0 for “Never experienced” and 1 for “Ever experienced” IPV during pregnancy. This recoding allowed for clear differentiation between women who had experienced IPV during pregnancy and those who had not.

### Covariates

The independent variables husband’s age group was divided into four categories, including 15–29 years, 30–44 years, 45–59 years, and 60 years and above. The residence variable was categorized into rural and urban groups. Additionally, the wealth index was classified into three categories: middle, poor, and rich. The original wealth index variable from the DHS had five categories, but due to missing values, the “poorest” and “poorer” categories were combined into the “poor” group, while the “richer” and “richest” categories were grouped into the “rich” group, with the middle-class category remaining unchanged. Furthermore, the husband’s education status was encoded into four categories: no education, primary education, secondary education, and higher education. The husband’s working status was categorized as working or not working. Furthermore, the spouse’s educational gap was considered and categorized as follows: Spouse higher educated than the respondent (where the husband’s years of education were higher than the pregnant woman’s), spouse less educated than the respondent (where the husband’s years of education were lower than the pregnant woman’s), both highly educated (where the years of education for both were equal), and both uneducated (neither the husband nor the woman had any formal education).

### Weighting

Survey research often employs a technique called weighting to refine the collected data. This approach aims to enhance the accuracy of results and boost the reliability of survey estimates. In this manner, this can prevent any bias and obtain accurate estimates regarding the characteristics of the entire population. It is important to note that different units of analysis require different types of weights—households, women, men, children, couples—and the DHS Program employs the weighting class adjustment approach. This approach involves creating response groups and calculating a response rate within each group to adjust the design weight accordingly.

### Data analysis

For data analysis, Stata 17 software was utilized. Given the complex survey design of the DHS dataset, adjustments were made to account for sampling weights, stratification, and clustering to reduce bias and improve estimate accuracy. After thoroughly analyzing the DHS dataset, the demographic characteristics of all factors were summarized for each country. To investigate the association between each covariate and the dependent variable, a chi-square test was performed for each available country. The proportion of each covariate with the outcome variable was assessed, and its significance was identified. Chi-square significance was labeled with (Ψ) to indicate the strength of the significance level.

To adjust for potential confounders and determine the independent effect of each variable on the outcomes of interest, multivariate logistic regression was employed. All independent variables with a minimum p-value ≤ 0.2 in the chi-square test were included in the multivariate model, ensuring that all relevant variables were appropriately fitted. Binary logistic regression was used to calculate adjusted odds ratios (AOR) and 95% confidence intervals (CI). Each odds ratio was assessed for significance, with star (*) values indicating the strength of significance.

Additional analyses, including multi-collinearity checks and pairwise correlation assessments, were performed to detect any multi-collinearity among variables, but none was found. The odds ratios, confidence intervals, and p-values for each covariate were interpreted in relation to the research question. Finally, data and results were presented using tables to effectively summarize and visualize the key findings.

## Results

**Fig. 1:**
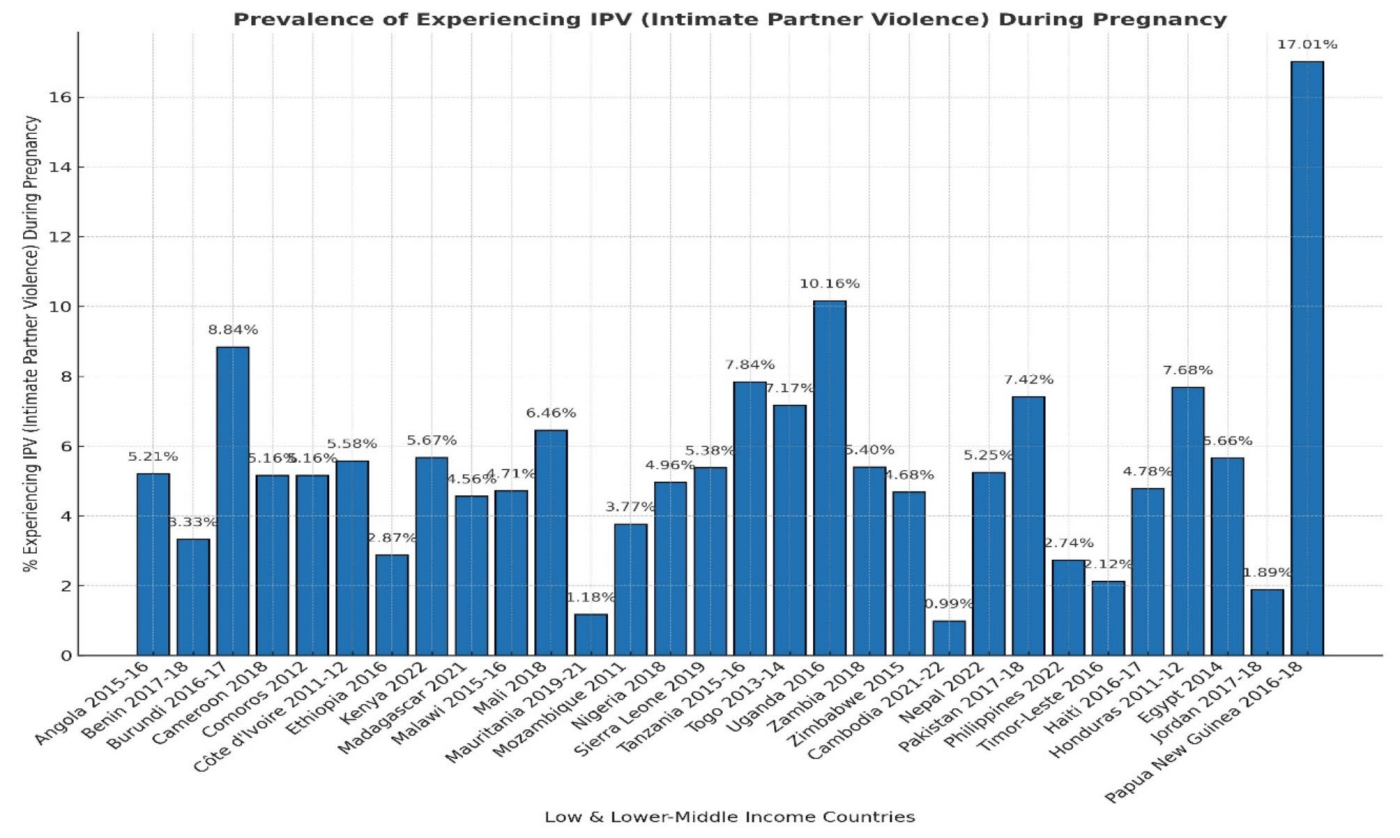
Bar chart showing the Prevalence of IPV during pregnancy among Low and LMIC countries.

The study encompassed 152,643 pregnant women across all five continents. Among them, 8357 experienced IPV during pregnancy. Papua New Guinea, a country in the Oceania region, shows (Fig. 1) the highest percentage of IPV against pregnant women (17.01%; 95% CI 15.76–18.38%), whereas Cambodia, a country in South and Southeast Asia, shows the lowest (0.99%; 95% CI 0.88–1.10%). Overall, IPV during pregnancy across the five continents was (5.47%; 95% CI 5.30–5.65%).

### Demographic factors influencing IPV in pregnant women

In Table 1, detailed demographic characteristics of individuals from low- and LMIC countries were presented. However, in Table 2, demographic factors, particularly the husband’s age group and residence exhibited strong associations with the risk of IPV during pregnancy across various regions. In sub-Saharan Africa, older husband age (45–59 years) was associated with a lower likelihood of IPV in Côte d’Ivoire (AOR: 0.41, CI 0.22–0.76, *P* < 0.001), Tanzania (AOR: 0.62, CI 0.44–0.88, *P* < 0.001), and Togo (AOR: 0.63, CI 0.42–0.96, *P* < 0.05), suggesting potential protective factors linked to emotional maturity or changing dynamics with age. However, in Ethiopia and Malawi, an opposing trend was observed, with a higher IPV risk in the same age group (AOR: 2.85, CI 1.10–7.34, *P* < 0.05) and (AOR: 1.96, CI 1.10–5.51, *P* < 0.05). Regarding residence, urban areas were associated with a higher risk of IPV in Angola (AOR: 2.47, CI 1.58–3.86, *P* < 0.05), Burundi (AOR: 1.42, CI 1.01–2.01, *P* < 0.05) and Malawi (AOR: 2.09, CI 1.20–3.67, *P* < 0.001), compared to rural areas.

**Table 1:** Demographic characteristics of individuals of low and LMIC countries.

Characteristics	Sub-Sahara Africa																			
	Angola	Benin	Burundi	Cameroon	Comoros	Cote d’Ivoire	Ethiopia	Kenya	Madagascar	Malawi	Mali	Mauritania	Mozambique	Nigeria	Sierra Leone	Tanzania	Togo	Uganda	Zambia	Zimbabwe
	*N* (%)	*N* (%)	*N* (%)	*N* (%)	*N* (%)	*N* (%)	*N* (%)	*N* (%)	*N* (%)	*N* (%)	*N* (%)	*N* (%)	*N* (%)	*N* (%)	*N* (%)	*N* (%)	*N* (%)	*N* (%)	*N* (%)	*N* (%)
Husband’s age group																				
15–29	1632 (32.08)	715 (18.93)	1586 (25.47)	601 (15.19)	288 (15.93)	533 (13.13)	728 (18.21)	1884 (18.98)	1377 (31)	1385 (31)	361 (12.59)	134 (7.62)	1351 (29.16)	816 (10.22)	476 (13.56)	1381 (21.99)	727 (15.89)	1652 (27.94)	1445 (24.95)	958 (20.58)
30–44	2427 (47.72)	2049 (54.22)	3270 (52.51)	2202 (55.72)	984 (54.52)	2245 (55.27)	2158 (53.96)	5625 (56.67)	2125 (48)	2244 (51)	1482 (51.77)	910 (51.47)	2236 (48.26)	4423 (55.41)	1709 (48.64)	3294 (52.51)	2491 (54.45)	2982 (50.45)	3155 (54.52)	2664 (57.26)
45–59	941 (18.51)	875 (23.15)	1163 (18.65)	902 (22.81)	393 (21.79)	1025 (25.24)	844 (21.12)	2143 (21.59)	825 (19)	646 (16)	768 (26.83)	599 (33.87)	915 (19.75)	2185 (27.37)	1035 (29.46)	1391 (22.17)	1146 (25.06)	1109 (18.76)	1099 (18.99)	898 (19.31)
60 and above	85 (1.69)	139 (3.70)	209 (3.37)	248 (6.28)	140 (7.76)	258 (6.36)	270 (6.71)	274 (2.76)	102 (2)		253 (8.80)	125 (7.04)	131 (2.83)	559 (7.00)	293 (8.34)	209 (3.33)	210 (4.60)	168 (2.85)	89 (1.54)	132 (2.85)
Residence																				
Rural area	1890 (37.18)	2288 (60.56)	5722 (91.88)	2034 (51.45)	1258 (69.69)	2454 (60.41)	3456 (86.40)	6152 (61.98)	3709 (84)	3695 (85)	2342 (81.80)	1000 (56.56)	3385 (73.06)	4294 (53.79)	2339 (66.58)	4402 (70.15)	2806 (61.34)	4623 (78.20)	3513 (60.70)	3092 (66.47)
Urban area	3195 (62.82)	1490 (39.44)	506 (8.12)	1919 (48.55)	547 (30.31)	1608 (39.59)	544 (13.60)	3774 (38.02)	720 (16)	674 (15)	522 (18.20)	768 (43.44)	1248 (26.94)	3689 (46.21)	1174 (33.42)	1873 (29.85)	1768 (38.66)	1288 (21.80)	2275 (39.30)	1560 (33.53)
Wealth index																				
Middle	1023 (20.12)	784 (20.75)	1305 (20.95)	740 (18.72)	366 (20.28)	781 (19.22)	836 (20.91)	1864 (18.78)	963 (22)	892 (20)	622 (21.70)	329 (18.61)	1056 (22.80)	1580 (19.79)	702 (19.97)	1242 (19.80)	963 (21.05)	1174 (19.86)	1108 (19.14)	812 (17.44)
Poor	2010 (39.52)	1413 (37.40)	2809 (45.11)	1685 (42.63)	778 (43.12)	1846 (45.43)	1680 (42.02)	3545 (35.72)	1729 (39)	1816 (42)	1215 (42.42)	770 (43.58)	2017 (43.53)	2925 (36.64)	1666 (47.42)	2456 (39.15)	1705 (37.29)	2457 (41.55)	2414 (41.71)	1852 (39.81)
Rich	2052 (40.36)	1581 (41.86)	2114 (33.93)	1528 (38.65)	661 (36.60)	1435 (35.34)	1484 (37.06)	4517 (45.50)	1737 (39)	1661 (38)	1027 (35.88)	669 (37.81)	1560 (33.67)	3478 (43.57)	1145 (32.61)	2576 (41.05)	1906 (41.66)	2280 (38.59)	2266 (39.15)	1988 (42.75)
Husband’s Education																				
No education	622 (12.23)	1899 (59.25)	2519 (40.42)	864 (21.85)	707 (39.15)	2192 (53.95)	1933 (48.34)	618 (6.23)	835 (19)	431 (10)	2177 (76.00)	1078 (60.96)	1061 (22.89)	2316 (29.01)	1938 (55.16)	766 (12.21)	1059 (23.15)	406 (6.87)	342 (5.91)	70 (1.52)
Primary	1585 (31.17)	911 (24.13)	3069 (49.28)	1268 (32.09)	400 (22.19)	926 (22.80)	1486 (37.16)	3878 (39.06)	2042 (46)	2432 (56)	265 (9.27)	334 (18.91)	2685 (57.97)	1144 (14.33)	294 (8.37)	4403 (70.17)	1404 (30.69)	3234 (54.71)	2173 (37.55)	1017 (21.85)
Secondary	2486 (48.89)	756 (20.02)	551 (8.85)	1446 (36.58)	467 (25.88)	759 (18.69)	351 (8.79)	3331 (33.56)	1404 (32)	1312 (30)	309 (10.82)	277 (15.68)	819 (17.68)	3169 (39.70)	965 (27.46)	939 (14.97)	1811 (39.59)	1583 (26.79)	2806 (48.48)	3116 (66.98)
Higher	392 (7.71)	212 (5.60)	89 (1.44)	375 (9.48)	231 (12.78)	185 (4.56)	230 (5.72)	2099 (21.15)	148 (3)	194 (4)	113 (3.91)	79 (4.45)	68 (1.46)	1354 (16.96)	316 (9.01)	167 (2.65)	300 (6.57)	688 (11.63)	467 (8.06)	449 (9.65)
Husband’s Work Status																				
Not Working	494 (9.70)	58 (1.56)	270 (4.33)	72 (1.82)	74 (4.10)	66 (1.61)	307 (7.67)	796 (8.02)	70 (2)	367 (8)	240 (8.38)	356 (20.14)	184 (3.98)	237 (2.97)	220 (6.27)	64 (1.02)	78 (1.70)	190 (3.22)	593 (10.23)	720 (15.47)
Working	4591 (90.30)	3720 (98.44)	5958 (95.67)	3881 (98.18)	1731 (95.90)	3996 (98.39)	3694 (92.33)	9130 (91.98)	4359 (98)	4002 (92)	2624 (91.62)	1412 (79.86)	4449 (96.02)	7746 (97.03)	3293 (93.73)	6211 (98.98)	4496 (98.30)	5721 (96.78)	5195 (89.77)	3932 (84.53)
Spouse education gap																				
Both are uneducated	510 (10.04)	1648 (3874)	1722 (27.66)	691 (17.46)	474 (26.23)	1839 (45.27)	1676 (41.89)	453 (4.57)	473 (11)	201 (5)	1857 (64.83)	646 (36.55)	803 (17.32)	2008 (25.15)	1553 (44.22)	373 (5.93)	807 (17.63)	217 (3.67)	139 (2.40)	13 (0.28)
Spouse higher educated than respondent	3398 (66.82)	1464 (38.74)	2432 (39.06)	1822 (46.09)	776 (42.97)	1520 (37.42)	554 (13.87)	4003 (40.33)	1844 (42)	2586 (59)	493 (17.21)	433 (24.48)	2688 (58.02)	2999 (37.57)	1245 (35.43)	2133 (34.00)	2817 (61.59)	3330 (56.33)	3495 (60.39)	2243 (48.21)
Spouse lower educated than respondent	674 (13.26)	527 (13.95)	1467 (23.56)	790 (20.00)	460 (25.53)	566 (13.93)	197 (4.82)	2352 (23.69)	1511 (34)	1028 (24)	413 (14.45)	605 (34.22)	739 (15.96)	1064 (13.33)	548 (15.61)	1217 (19.39)	679 (14.85)	1562 (26.43)	1205 (20.81)	988 (21.25)
Both are highly educated	503 (9.88)	138 (3.67)	607 (9.73)	650 (16.45)	95 (5.27)	137 (3.38)	193 (4.82)	3118 (31.41)	602 (14)	554 (12)	101 (3.51)	84 (4.75)	403 (8.70)	1912 (23.95)	167 (4.74)	2552 (40.68)	271 (5.93)	802 (13.57)	949 (16.40)	1408 (30.26)

Characteristics	South and Southeast Asia					Latin America & Caribbean		*N*. Africa & West Asia		Oceania										
	Cambodia	Nepal	Pakistan	Philippines	Timor-Leste	Haiti	Honduras	Egypt	Jordan	Papua New Guinea										
	*N* (%)	*N* (%)	*N* (%)	*N* (%)	*N* (%)	*N* (%)	*N* (%)	*N* (%)	*N* (%)	*N* (%)										
Husband’s Age Group																				
15–29	925 (18.45)	831 (21.51)	582 (18.93)	1875 (16.79)	635 (18.25)	639 (17.86)	2820 (29.18)	966 (16.00)	616 (10.60)	649 (20.30)										
30–44	3218 (64.20)	2196 (56.84)	1597 (52.00)	6482 (58.03)	1880 (54.08)	1946 (54.38)	4850 (50.18)	3195 (52.78)	3074 (52.90)	1837 (57.46)										
45–59	825 (16.46)	799 (20.69)	830 (27.02)	2683 (24.02)	865 (24.88)	860 (24.05)	1769 (18.31)	1740 (28.75)	1994 (34.32)	671 (20.98)										
60 and above	45 (0.89)	37 (0.96)	63 (2.05)	130 (1.16)	97 (2.78)	132 (3.71)	227 (2.33)	153 (2.47)	126 (2.17)	41 (1.26)										
Residence																				
Rural area	3131 (62.46)	1306 (33.79)	1139 (37.08)	5222 (46.75)	2829 (75.60)	2221 (62.11)	5138 (53.16)	3940 (65.09)	594 (10.22)	2899 (90.65)										
Urban area	1882 (37.54)	2557 (66.21)	1139 (37.08)	5948 (53.25)	848 (24.40)	1356 (37.89)	4528 (46.84)	2114 (34.91)	5216 (89.78)	299 (9.35)										
Wealth index																				
Middle	975 (19.45)	788 (20.39)	602 (19.60)	2309 (20.67)	746 (21.46)		2009 (20.78)	1426 (23.55)	1229 (21.15)	678 (21.21)										
Poor	1906 (38.03)	1559 (40.35)	1219 (39.70)	4833 (43.27)	1455 (41.85)	692 (19.34)	3876 (40.11)	2167 (35.79)	2341 (40.30)	1315 (41.11)										
Rich	2132 (42.52)	1516 (39.26)	1251 (40.70)	4028 (36.06)	1276 (36.69)	1474 (41.20)	3781 (39.11)	2461 (40.66)	2240 (38.55)	1205 (37.68)										
Husband’s Education						1411 (39.47)														
No education	470 (9.38)	549 (14.22)	938 (30.55)	126 (1.13)	976 (28.06)	638 (17.84)	613 (6.34)	901 (14.88)	118 (2.04)	654 (20.46)										
Primary	2026 (40.42)	1620 (41.93)	510 (16.61)	1327 (11.88)	744 (21.38)	1186 (33.15)	6257 (64.73)	870 (14.37)	547 (9.42)	1470 (45.96)										
Secondary	2157 (43.03)	1424 (36.86)	993 (32.31)	5888 (52.71)	1370 (39.42)	1463 (40.89)	2245 (23.23)	3271 (54.03)	3343 (57.53)	841 (26.29)										
Higher	360 (7.17)	270 (6.99)	631 (20.53)	3829 (34.28)	387 (11.14)	290 (8.12)	551 (5.70)	1012 (16.72)	1802 (31.010)	233 (7.29)										
Husband’s Work Status																				
Not working	142 (2.83)	79 (2.04)	111 (3.62)	521 (4.66)	795 (22.85)	182 (5.09)	15 (0.15)	172 (2.85)	1046 (18.00)	1609 (50.30)										
Working	4871 (97.17)	3784 (97.96)	2961 (96.38)	10,649 (95.34)	2682 (77.15)	3395 (94.91)	9651 (99.85)	5882 (97.15)	4765 (82.00)	1589 (49.70)										
Spouse education gap																				
Both are uneducated	234 (4.66)	440 (11.39)	778 (25.32)	48 (0.43)	635 (18.25)	372 (10.39)	222 (2.29)	606 (10.01)	45 (0.77)	412 (12.90)										
Spouse higher educated than respondent	2507 (50.02)	2039 (52.78)	1419 (46.18)	3973 (35.57)	1277 (36.73)	1864 (52.11)	3124 (32.32)	2524 (41.69)	1773 (30.51)	1502 (46.96)										
Spouse lower educated than respondent	1507 (30.06)	847 (21.93)	522 (17.00)	3767 (33.72)	934 (26.88)	931 (12.01)	4065 (42.06)	1461 (24.13)	1680 (28.95)	791 (24.73)										
Both are highly educated	765 (15.26)	537 (13.90)	353 (11.50)	3382 (30.28)	631 (18.13)	430 (12.01)	2255 (23.33)	1463 (24.17)	1682 (28.95)	493 (15.41)										

**Table 2:** Associations and influences of factors towards women experiencing IPV during pregnancy across low and LMIC countries.

Characteristics	Sub-Sahara Africa														
	Angola			Benin			Burundi			Cameroon			Comoros		
	*N* (%)	AOR	95% CI	*N* (%)	AOR	95% CI	*N* (%)	AOR	95% CI	*N* (%)	AOR	95% CI	*N* (%)	AOR	95% CI
Husband’s Age Group	X^2^ = 5.06			X^2^ = 1.54			X^2^ = 4.02			X^2^ = 0.15			X^2^ = 5.64		
15–29	84 (31.82)	Ref		23 (17.98)	Ref		132 (24.05)	Ref		33 (15.93)	Ref		10 (19.30)	Ref	
30–44	137 (51.91)	1.11	0.79–1.56	72 (57.24)	1.24	0.73–2.11	291 (52.83)	1.01	0.84–1.41	114 (55.93)	1.02	0.65–1.58	29 (58.00)	0.94	0.40–2.19
45–59	30 (11.40)	0.73	0.44–1.20	23 (18.25)	1.19	0.66–2.14	114 (20.85)	1.22	0.89–1.68	45 (22.20)	1.03	0.62–1.70	5 (10.00)	0.31	0.09–1.10
60 and above	12 (4.56)	1.38	0.48–3.99	8 (6.34)	0.70	0.14–3.18	14 (2.28)	0.65	0.33–1.31	12 (5.93)	1.06	0.52–2.18	6 (11.92)	1.39	0.25–7.46
Residence	X^2^ = 4.99			X^2^ = 11.37^ΨΨΨ^			X^2^ = 0.27			X^2^ = 4.52			X^2^ = 1.2		
Rural area	81 (30.62)	Ref		95 (74.94)	Ref		510 (92.47)	Ref		120 (58.89)	Ref		31 (62.43)	Ref	
Urban area	184 (69.38)	2.47	1.58–3.86*	31 (25.06)	0.48	0.30–0.78	41 (7.53)	1.42	1.01–2.01*	84 (41.11)	0.74	0.45–1.22	19 (37.57)	1.58	0.72–3.43
Wealth index	X^2^ = 13.41^Ψ^			X^2^ = 3.56			X^2^ = 10.04^Ψ^			X^2^ = 4.98			X^2^ = 1.53		
Middle	75 (28.11)	Ref		34 (26.78)	Ref		118 (21.44)	Ref		47 (23.06)	Ref		7 (14.01)	Ref	
Poor	106 (40.03)	1.14	0.69–1.89	48 (37.69)	0.87	0.53–1.40	279 (50.60)	1.09	0.84–1.40	93 (45.29)	0.91	0.54–1.53	21 (42.67)	1.52	0.65–3.51
Rich	85 (31.87)	0.56	0.36–0.85**	44 (35.53)	0.73	0.43–1.24	154 (27.95)	0.81	0.59–1.12	65 (31.65)	0.69	0.42–1.14	22 (43.32)	2.03	062 − 6.60
Husband’s Education	X^2^ = 5.46			X^2^ = 5.36			X^2^ = 16.61^ΨΨΨ^			X^2^ = 8.29			X^2^ = 3.42		
No education	37 (14.07)	Ref		56 (43.99)	Ref		232 (42.10)	Ref		34 (16.69)	Ref		18 (35.65)	Ref	
Primary	93 (35.04)	1.15	0.34–3.91	41 (32.17)	1.89	0.77–4.63	280 (50.83)	0.74	0.48–1.13	82 (40.12)	2.98	1.20–7.41*	13 (26.35)	0.65	0.12–3.31
Secondary	122 (46.09)	0.89	0.25–3.22	25 (24.27)	1.80	0.63–5.11	30 (5.36)	0.40	0.22–0.74***	75 (36.51)	3.18	1.23–8.22*	11 (22.00)	0.64	0.16–2.45
Higher	13 (4.80)	0.65	0.15–2.81	6 (5.82)	1.18	0.28–4.81	9 (1.63)	0.05	0.01–0.41	14 (6.67)	2.86	0.90–9.09	8 (16.00)	0.16	0.02–1.02
Husband’s Work Status	X^2^ = 1.02			X^2^ = 2.07			X^2^ = 0.42			X^2^ = 0.37			X^2^ = 0.27		
Not Working	21 (7.88)	Ref		0 (0.00)	Ref		21 (3.78)	Ref		6 (2.87)	Ref		2 (2.59)	Ref	
Working	244 (92.12)	1.39	0.82–2.36	126 (100)	Not available		530 (96.22)	1.09	0.64–1.85	203 (97.13)	1.46	0.38–5.57	49 (97.41)	1.92	0.36–2.12
Spouse education gap	X^2^ = 1.15			X^2^ = 7.63			X^2^ = 4.87			X^2^ = 7.29			X^2^ = 0.72		
Both are uneducated	31.5 (11.87)	Ref		46 (35.93)	Ref		161 (29.21)	Ref		29 (14.05)	Ref		11 (22.00)	Ref	
Spouse higher educated than respondent	174 (65.56)	0.77	0.20–2.90	49 (38.34)	0.82	0.30–2.23	223 (40.63)	1.57	0.97–2.56	83 (40.81)	0.43	0.15–1.24	19 (38.00)	2.20	0.34–1.29
Spouse lower educated than respondent	36 (13.49)	0.74	0.23–2.41	20 (15.87)	1.66	0.76–3.62	126 (23.00)	1.11	0.78–1.55	53 (26.15)	0.83	0.33–2.10	12 (24.00)	1.11	0.24–5.05
Both are highly educated	24 (9.09)	0.69	0.16–3.06	11 (8.73)	1.03	0.28–3.80	41 (7.16)	1.06	0.04–0.15	39 (18.99)	0.61	0.22–1.73	8 (16.00)	1.76	0.21–4.50

Characteristics	Sub-Sahara Africa														
	Cote d’Ivoire			Ethiopia			Kenya			Madagascar			Malawi		
	*N* (%)	AOR	95% CI	*N* (%)	AOR	95% CI	*N* (%)	AOR	95% CI	*N* (%)	AOR	95% CI	*N* (%)	AOR	95% CI
Husband’s Age Group	X^2^ = 17.13^Ψ^			X^2^ = 14.33			X^2^ = 9.76			X^2^ = 10.53^Ψ^			X^2^ = 9.86		
15–29	47 (19.97)	Ref		12 (10.11)	Ref		87 (15.52)	Ref		76 (37.57)	Ref		42 (20.38)	Ref	
30–44	137 (57.75)	0.68	0.42–1.08	60 (52.91)	1.71	0.74–3.94	341 (60.51)	1.29	0.93–1.80	99 (48.80)	0.92	0.61–1.38	118 (57.06)	1.59	1.02–2.50
45–59	39 (16.37)	0.41	0.22–0.76***	35 (30.70)	2.85	1.10–7.34*	112 (19.95)	1.02	0.71–1.47	26 (13.63)	0.68	0.40–1.14	38 (18.61)	1.96	1.10–3.51*
60 and above	14 (5.90)	0.62	0.29–1.32	7 (6.14)	0.67	0.15–2.99	23 (4.01)	1.70	0.97–2.98	0 (0.00)	Not available		8 (3.88)	0.60	0.14–2.66
Residence	X^2^ = 3.21			X^2^ = 0.52			X^2^ = 7.31			X^2^ = 5.15^Ψ^			X^2^ = 10.77^Ψ^		
Rural area	130 (54.88)	Ref		102 (88.82)	Ref		379 (67.27)	Ref		158 (77.97)	Ref		158 (76.52)	Ref	
Urban area	107 (45.12)	1.31	0.72–2.41	12 (11.18)	1.20	0.48–3.09	184 (32.73)	1.13	0.82–1.55	43 (22.03)	1.43	0.96–2.13	48 (23.48)	2.09	1.20–3.67**
Wealth index	X^2^ = 2.51			X^2^ = 2.19			X^2^ = 27.11^ΨΨΨ^			X^2^ = 3.77			X^2^ = 8.87		
Middle	42 (17.71)	Ref		21 (17.89)	Ref		110 (19.57)	Ref		51 (25.34)	Ref		25 (12.25)	Ref	
Poor	100 (42.17)	1.17	0.62–2.23	56 (49.07)	1.37	0.61–3.03	252 (44.71)	1.18	0.89–1.55	66 (32.77)	0.88	0.57–1.37	95 (46.02)	2.08	1.13–3.81*
Rich	95 (40.12)	1.17	0.69–1.97	37 (33.04)	1.18	0,46-3.01	201 (35.72)	0.90	0.63–1.29	84 (41.90)	0.71	0.47–1.09	86 (41.73)	1.53	0.79–2.96
Husband’s Education	X^2^ = 11.66			X^2^ = 7.79			X^2^ = 78.88^ΨΨΨ^			X^2^ = 14.63^ΨΨΨ^			X^2^ = 1.19		
No education	107 (45.05)	Ref		60 (52.63)	Ref		29 (5.07)	Ref		19 (9.56)	Ref		16 (7.98)	Ref	
Primary	69 (29.39)	2.58	1.17–5.67*	38 (32.97)	0.53	0.13–2.13	296 (52.55)	1.46	0.72–2.96	94 (46.53)	1.11	0.59–2.13	120 (58.14)	1.36	0.60–3.12
Secondary	53 (22.50)	2.44	1.05–5.67*	6 (5.63)	0.38	0.07–1.98	192 (34.18)	1.07	0.52–2.21	81 (40.28)	1.49	0.70–3.20	60 (29.02)	1.19	0.46–3.14
Higher	8 (3.06)	1.25	0.38–4.07	10 (8.77)	0.11	0.02–0.67	46 (8.20)	0.37	0.17–0.85*	7 (3.62)	1.24	0.42–3.70	10 (4.86)	0.96	0.27–3.48
Husband’s Work Status	X^2^ = 11.45^Ψ^			X^2^ = 1.24			X^2^ = 1.88			X^2^ = 0.18			X^2^ = 0.68		
Not Working	10 (4.30)	Ref		12 (10.15)	Ref		37 (6.52)	Ref		7 (1.94)	Ref		14 (6.85)	Ref	
Working	227 (95.70)	0.35	0.12–1.01	102 (89.45)	0.69	0.13–3.58	526 (93.48)	1.45	0.98–2.14	194 (98.06)	0.76	0.28–2.08	192 (93.15)	1.20	0.64–2.23
Spouse education gap	X^2^ = 10.04			X^2^ = 3.75			X^2^ = 17.57^Ψ^			X^2^ = 24.79^ΨΨΨ^			X^2^ = 5.48		
Both are uneducated	88 (37.18)	Ref		58 (50.45)	Ref		19 (3.53)	Ref		10 (4.97)	Ref		7 (3.25)	Ref	
Spouse higher educated than respondent	96 (40.40)	0.50	0.20–1.25	31 (27.19)	1.51	0.32–7.18	254 (45.15)	1.57	0.74–3.34	80 (39.80)	5.71	3.35–7.65	129 (62.60)	1.32	0.47–3.72
Spouse lower educated than respondent	46 (19.52)	1.04	0.55–1.97	16 (13.99)	1.26	0.43–3.65	152 (26.98)	1.17	0.58–2.36	85 (42.16)	2.49	1.60–3.32	54 (26.07)	1.46	0.59–3.64
Both are highly educated	7 (2.90)	0.41	0.12–1.41	9 (7.89)	2.21	0.27–7.93	117 (3.53)	0.90	0.42–1.94	26 (12.69)	1.07	1.91–3.06	17 (8.09)	0.69	0.20–2.38

Characteristics	Sub-Sahara Africa														
	Mali			Mauritania			Mozambique			Nigeria			Sierra Leone		
	*N* (%)	AOR	95% CI	*N* (%)	AOR	95% CI	*N* (%)	AOR	95% CI	*N* (%)	AOR	95% CI	*N* (%)	AOR	95% CI
Husband’s Age Group	X^2^ = 6.59			X^2^ = 0.58			X^2^ = 4.62			X^2^ = 7.09			X^2^ = 9.36		
15–29	20 (10.67)	Ref		2 (9.24)	Ref		51 (29.44)	Ref		50 (12.69)	Ref		24 (12.70)	Ref	
30–44	113 (60.95)	1.56	0.83–2.95	11 (55.42)	0.80	0.12–5.20	95 (54.45)	1.07	0.73–1.58	226 (57.04)	0.84	0.56–1.24	111 (58.93)	1.41	0.82–2.42
45–59	40 (22.07)	1.05	0.52–2.08	7 (32.11)	0.77	0.11–5.22	23 (13.63)	0.65	0.34–1.23	103 (25.91)	0.81	0.53–1.19	42 (22.49)	0.86	0.48–1.55
60 and above	12 (6.30)	0.90	0.40–2.05	1 (3.23)	0.33	0.02–4.78	5 (2.48)	0.87	0.30–2.53	17 (4.36)	0.59	0.33–1.07	11 (5.88)	0.84	0.40–1.77
Residence	X^2^ = 1.83			X^2^ = 3.38^Ψ^			X^2^ = 9.71^ΨΨΨ^			X^2^ = 0.00			X^2^ = 5.85^Ψ^		
Rural area	159 (85.59)	Ref		16 (76.60)	Ref		109 (62.41)	Ref		213 (53.70)	Ref		141 (74.58)	Ref	
Urban area	26 (14.41)	0.67	0.36–1.23	5 (23.40)	0.64	0.22–1.84	66 (37.59)	1.26	0.80–1.98	184 (46.30)	0.91	0.67–1.24	48 (25.42)	0.85	0.52–1.39
Wealth index	X^2^ = 0.57			X^2^ = 5.13			X^2^ = 16.88^ΨΨΨ^			X^2^ = 1.39			X^2^ = 11.30^Ψ^		
Middle	38 (20.81)	Ref		6 (29.55)	Ref		31 (17.72)	Ref		80 (20.13)	Ref		52 (27.50)	Ref	
Poor	83 (45.14)	1.20	0.78–1.85	12 (56.24)	0.83	0.27–2.57	59 (33.66)	Not available	0.61–1.64	134 (33.91)	1.09	0.76–1.60	93 (49.20)	0.73	0.47–1.12
Rich	64 (34.05)	1.17	0.68–2.03	3 (14.21)	0.37	0.13–1.05	85 (48.61)	1.81	1.08–3.01*	182 (45.95)	1.04	0.73–1.48	44 (23.31)	0.52	0.31–0.87*
Husband’s Education	X^2^ = 5.96			X^2^ = 2.43			X^2^ = 10.31^Ψ^			X^2^ = 34.00^ΨΨΨ^			X^2^ = 2.30		
No education	132 (71.35)	Ref		15 (70.80)	Ref		23 (13.10)	Ref		78 (19.68)	Ref		96 (50.97)	Ref	
Primary	22 (11.98)	2.58	1.12–5.94*	5 (22.43)	0.13	0.02–1.02	113 (64.67)	1.41	0.60–3.32	62 (15.63)	1.79	0.70–4.57	19 (10.01)	0.84	0.27–2.56
Secondary	24 (13.12)	2.99	1.23–7.7.24*	1 (6.77)	0.04	0.00-0.65	34 (19.60)	0.94	0.36–2.47	207 (52.22)	2.32	0.91–5.95	58 (30.84)	0.76	0.25–2.29
Higher	7 (3.78)	0.83	0.19–3.53	0 (0.00)	Not available		5 (2.62)	1.35	0.39–4.64	49 (12.46)	1.31	0.47–3.59	15 (8.19)	0.72	0.20–2.52
Husband’s Work Status	X^2^ = 0.13			X^2^ = 0.24			X^2^ = 0.25			X^2^ = 4.61^Ψ^			X^2^ = 1.18		
Not Working	17 (9.12)	Ref		3 (15.79)	Ref		6 (3.23)	Ref		5 (1.17)	Ref		8 (4.42)	Ref	
Working	168 (90.88)	0.81	0.42–1.58	17 (84.21)	1.51	0.50–4.56	169 (96.77)	1.18	0.45–3.08	392 (98.83)	2.25	0.94–5.38	181 (95.58)	1.17	0.53–2.61
Spouse education gap	X^2^ = 3.75			X^2^ = 6.11			X^2^ = 8.35^Ψ^			X^2^ = 16.85^ΨΨΨ^			X^2^ = 3.44		
Both are uneducated	115 (62.17)	Ref		3 (16.67)	Ref		17 (9.73)	Ref		66 (16.76)	Ref		77 (40.63)	Ref	
Spouse higher educated than respondent	27 (14.72)	0.39	0.16–0.97*	6 (27.55)	4.72	3.79–5.09	117 (66.85)	1.31	0.50–3.46	166 (41.85)	0.92	0.36–2.33	79 (41.56)	1.85	0.62–5.54
Spouse lower educated than respondent	25 (18.88)	1.09	0.64–1.84	12 (55.78)	5.20	1.84–4.65	25 (14.05)	0.95	0.40–2.25	66 (16.55)	1.21	0.52–2.75	25 (13.37)	0.99	0.58–1.72
Both are highly educated	8 (4.23)	0.52	0.15–1.74	0 (0.00)	Not available		16 (9.37)	1.10	0.37–3.26	98 (24.83)	0.79	0.31–2.07	8 (4.43)	1.59	0.42–6.07

Characteristics	Sub-Sahara Africa														
	Tanzania			Togo			Uganda			Zambia			Zimbabwe		
	*N* (%)	AOR	95% CI	*N* (%)	AOR	95% CI	*N* (%)	AOR	95% CI	*N* (%)	AOR	95% CI	*N* (%)	AOR	95% CI
Husband’s Age Group	X^2^ = 13.92^Ψ^			X^2^ = 6.23			X^2^ = 5.64			X^2^ = 13.13^Ψ^			X^2^ = 10.52^Ψ^		
15–29	129 (26.21)	Ref		62 (18.88)	Ref		160 (26.70)	Ref	75 (23.78)	75 (23.78)	Ref		64 (29.22)	Ref	
30–44	256 (51.92)	0.85	0.65–1.12	187 (57.08)	0.88	0.63–1.24	315 (52.46)	1.21	0.97–1.52	199 (62.97)	1.28	0.83–1.96	108 (49.81)	0.62	0.42–0.91
45–59	84 (17.07)	0.62	0.44–0.88***	66 (20.13)	0.63	0.42–0.96*	116 (19.38)	1.23	0.93–1.63	35 (11.07)	0.67	0.42–1.07	41 (18.66)	0.67	0.40–1.11
60 and above	24 (4.80)	1.07	0.62–1.86	13 (3.90)	0.56	0.30–1.04	9 (1.46)	0.58	0.29–1.16	7 (2.21)	0.75	0.24–2.37	5 (2.31)	0.48	0.16–1.40
Residence	X^2^ = 34.39^ΨΨΨ^			X^2^ = 1.12			X^2^ = 15.67^ΨΨΨ^			X^2^ = 0.00			X^2^ = 1.17		
Rural area	403 (81.82)	Ref		210 (64.09)	Ref		508 (84.46)	Ref		192 (60.68)	Ref		152 (69.89)	Ref	
Urban area	90 (18.18)	0.81	0.56–1.16	118 (35.91)	1.36	0.70–2.66	93 (15.54)	0.99	0.73–1.35	124 (39.32)	1.01	0.69–1.49	66 (30.11)	0.96	0.50–1.82
Wealth index	X^2^ = 57.86^ΨΨΨ^			X^2^ = 3.83			X^2^ = 73.78^ΨΨΨ^			X^2^ = 9.44			X^2^ = 4.36		
Middle	117 (23.85)	Ref		74 (22.61)	Ref		112 (18.60)	Ref		40 (12.70)	Ref		32 (14.76)	Ref	
Poor	252 (51.25)	1.03	0.76–1.39	134 (40.84)	1.01	0.74–1.39	342 (56.85)	1.58	1.24–2.01***	148 (46.89)	1.68	1.16–2.43***	101 (46.59)	1.33	0.81–2.19
Rich	123 (24.90)	0.60	0.42–0.87***	120 (36.54)	0.71	0.35–1.42	147 (24.54)	0.75	0.55–1.03	128 (40.41)	1.83	1.18–2.82***	84 (38.65)	1.38	0.67–2.84
Husband’s Education	X^2^ = 23.41^ΨΨΨ^			X^2^ = 12.62^Ψ^			X^2^ = 41.65^ΨΨΨ^			X^2^ = 11.37			X^2^ = 8.78		
No education	82 (16.75)	Ref		89 (27.18)	Ref		38 (6.31)	Ref		20 (6.19)	Ref		7 (3.00)	Ref	
Primary	351 (71.26)	0.75	0.46–1.24	115 (35.16)	0.99	0.54–1.81	390 (64.92)	1.18	0.68–2.03	132 (41.79)	1.12	0.57–2.22	57 (26.18)	0.52	0.18–1.54
Secondary	52 (10.56)	0.58	0.30–1.13	112 (34.23)	0.71	0.39–1.30	143 (23.83)	0.99	0.54–1.80	154 (48.83)	0.99	0.39–2.49	122 (57.81)	0.39	0.13–1.24
Higher	7 (1.42)	0.11	0.03–0.41	11 (3.43)	0.42	0.17–1.05	30 (4.95)	0.49	0.24–1.05	10 (3.19)	0.33	0.11–1.01	32 (16.01)	0.21	0.05–0.80*
Husband’s Work Status	X^2^ = 2.09			X^2^ = 4.46			X^2^ = 0.00			X^2^ = 2.29			X^2^ = 0.58		
Not Working	8 (1.65)	Ref		10 (3.15)	Ref		19 (3.17)	Ref		40 (12.75)	Ref		30 (13.61)	Ref	
Working	484 (98.35)	0.58	0.26–1.31	317 (96.85)	0.39	0.18–0.85*	582 (96.83)	1.14	0.68–1.90	276 (87.25)	0.79	0.42–1.48	188 (86.39)	1.35	0.87–2.10
Spouse education gap	X^2^ = 18.96^ΨΨΨ^			X^2^ = 4.06			X^2^ = 1.76			X^2^ = 2.66			X^2^ = 8.53		
Both are uneducated	34 (6.85)	Ref		68 (20.66)	Ref		17 (2.86)	Ref		8 (2.38)	Ref		8 (3.66)	Ref	
Spouse higher educated than respondent	188 (38.28)	1.86	0.90–3.83	187 (57.17)	0.94	0.48–1.81	345 (57.42)	1.61	0.79–3.24	179 (56.74)	0.99	0.33–3.03	110 (50.45)	1.12	0.10–2.59
Spouse lower educated than respondent	115 (23.39)	1.51	0.80–2.83	55 (16.81)	0.96	0.58–1.57	162 (26.97)	1.52	0.78–2.97	77 (24.28)	1.15	0.45–2.97	53 (24.22)	0.93	0.09–9.91
Both are highly educated	155 (31.48)	1.12	0.55–2.26	18 (5.36)	0.82	0.35–1.92	77 (12.75)	1.58	0.74–3.39	52 (16.61)	1.05	0.36–3.08	47 (21.55)	0.64	0.06–7.41

Characteristics	South and Southeast Asia														
	Cambodia			Nepal			Pakistan			Philippines			Timor-Leste		
	*N* (%)	AOR	95% CI	*N* (%)	AOR	95% CI	*N* (%)	AOR	95% CI	*N* (%)	AOR	95% CI	*N* (%)	AOR	95% CI
Husband’s Age Group	X^2^ = 5.06			X^2^ = 0.15			X^2^ = 9.76			X^2^ = 10.53^Ψ^			X^2^ = 8.55		
15–29	9 (17.38)	Ref		43 (21.12)	Ref		32 (14.01)	Ref		52 (16.77)	Ref		19 (25.92)	Ref	
30–44	30 (58.82)	1.16	0.44-3.00	113 (55.50)	0.99	0.68–1.43	114 (50.22)	1.41	0.83–2.38	174 (56.66)	0.97	0.63–1.49	36 (48.43)	0.63	0.33–1.19
45–59	6 (11.93)	0.76	0.23–2.52	43 (21.60)	0.95	0.56–1.62	76 (33.49)	1.74	0.98–3.09	77 (24.91)	0.99	0.53–1.87	13 (18.63)	0.51	0.21–1.21
60 and above	6 (11.76)	0.39	0.04–3.72	5 (1.78)	1.11	0.29–4.27	5 (2.28)	1.02	0.33–3.10	5 (1.67)	1.43	0.52–3.95	5 (6.97)	1.97	0.45–8.50
Residence	X^2^ = 4.99			X^2^ = 4.52			X^2^ = 7.31			X^2^ = 5.15^Ψ^			X^2^ = 0.31		
Rural area	31 (62.21)	Ref		79 (38.98)	Ref		160 (70.41)	Ref		144 (46.69)	Ref		58 (78.40)	Ref	
Urban area	19 (37.79)	1.65	0.87–3.15	124 (61.02)	1.01	0.70–1.46	67 (29.59)	0.93	0.60–1.44	164 (53.31)	1.24	0.89–1.73	15 (21.60)	0.70	0.33–1.48
Wealth index	X^2^ = 13.41^Ψ^			X^2^ = 4.98			X^2^ = 27.11^ΨΨΨ^			X^2^ = 3.77			X^2^ = 1.05		
Middle	6 (11.69)	Ref		52 (25.47)	Ref		50 (21.78)	Ref		89 (28.99)	Ref		12 (16.55)	Ref	
Poor	28 (55.32)	2.29	0.89–5.89	111 (54.59)	1.02	0.67–1.54	113 (49.66)	0.96	0.58–1.58	159 (51.59)	0.72	0.48–1.08	33 (44.62)	1.33	0.67–2.64
Rich	17 (32.99)	1.31	0.40–4.31	40 (19.94)	0.49	0.28–0.84***	65 (28.56)	0.81	0.41–1.54	60 (19.41)	0.46	0.20–1.01	28 (38.83)	1.59	0.79–3.18
Husband’s Education	X^2^ = 5.46			X^2^ = 8.29			X^2^ = 78.88^ΨΨΨ^			X^2^ = 14.63^ΨΨΨ^			X^2^ = 1.09		
No education	8 (15.06)	Ref		48 (23.60)	Ref		95 (41.87)	Ref		5 (0.87)	Ref		20 (27.15)	Ref	
Primary	24 (47.05)	2.25	0.64–7.89	108 (53.41)	0.52	0.24–1.13	44 (19.19)	0.86	0.26–2.90	66 (21.52)	1.53	0.40–5.79	13 (17.84)	0.98	0.31–3.15
Secondary	12 (23.55)	0.87	0.25–3.07	41 (20.26)	0.25	0.11–0.56**	66 (29.11)	0.66	0.19–2.29	174 (57.10)	0.74	0.19–2.88	30 (40.86)	1.24	0.33–4.67
Higher	6 (11.76)	1.27	0.12–3.75	6 (2.74)	0.22	0.06–0.80	22 (9.82)	0.34	0.09–1.28	63 (20.51)	0.46	0.11–1.92	10 (14.16)	1.59	0.36–6.96
Husband’s Work Status	X^2^ = 1.02			X^2^ = 0.37			X^2^ = 1.88			X^2^ = 0.18			X^2^ = 0.66		
Not Working	0 (0.00)	Ref		10 (4.74)	Ref		19 (8.04)	Ref		7 (2.18)	Ref		19 (28.85)	Ref	
Working	50 (100.00)	Not available		193 (95.26)	0.34	0.15–0.74***	209 (91.96)	0.43	0.21–0.87*	301 (97.82)	1.98	0.73–5.34	54 (73.15)	0.81	0.41–1.58
Spouse education gap	X^2^ = 1.15			X^2^ = 7.29			X^2^ = 17.57^Ψ^			X^2^ = 24.79^ΨΨΨ^			X^2^ = 1.35		
Both are uneducated	6 (11.65)	Ref		36 (17.80)	Ref		83 (36.39)	Ref		9 (2.91)	Ref		13 (18.29)	Ref	
Spouse higher educated than respondent	24 (48.38)	0.29	0.06–1.43	108 (52.99)	1.86	0.77–4.50	93 (40.64)	1.08	0.31–3.73	115 (37.21)	7.01	0.63–7.75	31 (42.54)	1.04	0.25–4.22
Spouse lower educated than respondent	12 (24.00)	0.31	0.08–1.10	44 (21.45)	1.36	0.66–2.82	29 (12.61)	0.79	0.32–1.93	106 (34.34)	4.39	0.42–6.39	18 (24.74)	0.89	0.28–2.77
Both are highly educated	8 (16.00)	0.04	0.00-0.45	16 (7.75)	1.29	0.46–3.58	24 (10.36)	1.28	0.33–4.99	79 (25.86)	5.11	0.45–7.21	11 (14.43)	0.69	0.15–3.13

Characteristics	Latin America & Caribbean						North Africa & West Asia						Oceania		
	Haiti			Honduras			Egypt			Jordan			Papua New Guinea		
	*N* (%)	AOR	95% CI	*N* (%)	AOR	95% CI	*N* (%)	AOR	95% CI	*N* (%)	AOR	95% CI	*N* (%)	AOR	95% CI
Husband’s Age Group	X^2^ = 9.95			X^2^ = 20.85^ΨΨΨ^			X^2^ = 11.63			X^2^ = 7.15			X^2^ = 7.35		
15–29	45 (26.43)	Ref		235 (31.59)	Ref		76 (22.03)	Ref		14 (12.52)	Ref		128 (23.56)	Ref	
30–44	85 (49.55)	0.64	0.40–0.99*	344 (46.28)	0.84	0.67–1.05	168 (48.92)	0.61	0.42–0.88	47 (43.08)	0.66	0.28–1.58	311 (57.16)	0.83	0.61–1.12
45–59	33 (19.71)	0.54	0.27–1.05	131 (17.63)	0.85	0.65–1.11	88 (25.66)	0.51	0.35–0.75***	43 (39.56)	0.72	0.27–1.92	100 (18.73)	0.75	0.49–1.15
60 and above	8 (4.31)	0.76	0.32–1.75	33 (4.50)	1.75	1.07–2.86*	12 (3.39)	0.75	0.28–2.02	6 (4.83)	0.98	0.22–4.36	5 (0.56)	0.34	0.08–1.45
Residence	X^2^ = 0.10			X^2^ = 16.10^ΨΨΨ^			X^2^ = 0.01			X^2^ = 1.36			X^2^ = 0.63		
Rural area	108 (63.28)	Ref		343 (46.19)	Ref		224 (65.43)	Ref		8 (6.93)	Ref		489 (89.73)	Ref	
Urban area	63 (38.72)	1.26	0.68–2.34	400 (53.81)	1.72	1.35–2.20***	119 (34.57)	1.42	0.95–2.11	102 (93.07)	1.55	0.73–3.27	56 (10.27)	1.01	0.57–1.78
Wealth index	X^2^ = 3.96			X^2^ = 29.29^ΨΨΨ^			X^2^ = 3.81			X^2^ = 3.91			X^2^ = 15.01^Ψ^		
Middle	35 (20.16)	Ref		209 (28.13)	Ref		86 (25.01)	Ref		15 (13.62)	Ref		143 (26.33)	Ref	
Poor	81 (47.31)	1.21	0.60–2.42	287 (38.66)	0.78	0.60–1.03	135 (39.34)	0.92	0.65–1.30	49 (44.14)	1.26	0.55–2.87	188 (34.49)	0.66	0.44–1.01
Rich	55 (32.53)	0.78	0.39–1.53	247 (33.21)	0.65	0.50–0.84***	122 (35.65)	0.78	0.53–1.16	46 (42.24)	1.98	0.70–5.57	213 (39.19)	0.71	0.49–1.03
Husband’s Education	X^2^ = 2.43			X^2^ = 20.47^ΨΨΨ^			X^2^ = 30.18^ΨΨΨ^			X^2^ = 12.77			X^2^ = 12.31		
No education	34 (19.67)	Ref		54 (7.33)	Ref		70 (20.32)	Ref		5 (4.54)	Ref		97 (17.81)	Ref	
Primary	55 (32.22)	0.68	0.32–1.46	515 (69.36)	Not available	0.67–1.51	63 (18.49)	0.68	0.36–1.28	16 (14.24)	0.59	0.10–3.26	235 (43.23)	0.60	0.32–1.11
Secondary	73 (42.87)	0.74	0.32–1.71	134 (18.08)	0.69	0.43–1.11	185 (53.79)	0.45	0.23–0.86*	69 (62.79)	0.36	0.07–1.79	156 (28.70)	0.71	0.33–1.54
Higher	9 (5.25)	0.53	0.17–1.60	39 (5.23)	0.32	0.14–0.72***	25 (7.39)	0.18	0.08–0.39***	20 (18.43)	0.13	0.02–0.93*	56 (10.26)	1.06	0.39–2.87
Husband’s Work Status	X^2^ = 0.46			X^2^ = 0.28			X^2^ = 0.01			X^2^ = 23.04^ΨΨΨ^			X^2^ = 2.00		
Not Working	11 (6.20)	Ref		6 (0.80)	Ref		9 (2.73)	Ref		39 (35.20)	Ref		259 (47.49)	Ref	
Working	160 (93.80)	0.83	0.40–1.69	738 (99.19)	1.49	0.18–12.75	333 (97.27)	1.17	0.46–2.95	71 (64.80)	0.44	0.20–0.98*	286 (52.51)	1.02	0.71–1.47
Spouse education gap	X^2^ = 2.72			X^2^ = 18.83^ΨΨΨ^			X^2^ = 10.24			X^2^ = 8.91			X^2^ = 24.51^ΨΨΨ^		
Both are uneducated	18 (10.72)	Ref		26 (3.52)	Ref		46 (13.55)	Ref		7 (7.77)	Ref		37 (6.87)	Ref	
Spouse higher educated than respondent	97 (56.76)	1.53	0.59–3.95	273 (36.81)	0.79	0.47–1.33	156 (45.46)	1.80	0.86–3.75	41 (45.55)	7.74	3.56–8.39	271 (49.79)	2.89	1.25–6.69*
Spouse lower educated than respondent	41 (23.91)	1.15	0.53–2.52	306 (41.14)	0.56	0.34–0.92*	75 (21.82)	0.93	0.50–1.75	20 (19.68)	3.51	0.28–3.56	159 (29.11)	3.12	1.58–6.14***
Both are highly educated	15 (8.61)	1.05	0.35–3.11	138 (18.54)	0.47	0.27–0.82***	66 (19.18)	1.27	0.58–2.79	22 (19.68)	3.53	0.24–5.84	77 (14.23)	2.58	1.14–5.85*

Denote: (N (%) = Number (percentage) of IPV-experienced women during pregnancy categorized by factors, *P-*value indication: * = *P-*value < 0.05, ** = *P-*value < 0.01, *** = *P-*value < 0.001, Chi^2^ significance: ^Ψ^ = *P-*value < 0.05, ^ΨΨ^ = *P-*value < 0.01, ^ΨΨΨ^ = *P-*value < 0.001, AOR = adjusted odds ratio)

In the Latin American region, specifically in Honduras, husbands aged 60 years and above were associated with a higher risk of IPV (AOR: 1.75, CI 1.07–2.86, *P* < 0.05), with urban residents also showing a slightly elevated IPV risk compared to rural residents (AOR: 1.72, CI 1.35–2.20, *P* < 0.001). In North Africa and West Asia, the age group 45–59 years was observed to have a lower risk of IPV in Egypt (AOR: 0.51, CI 0.35–0.75, *P* < 0.001), aligning with trends in sub-Saharan Africa for the 45–59 age group. Residence did not significantly influence IPV risk in this region. In South and Southeast Asia and Oceania, neither age nor residence showed a significant association with IPV.

### Socioeconomic factors

Socioeconomic factors also played a substantial role in influencing IPV victimization across regions. In sub-Saharan Africa, women from rich households had a lower risk of experiencing IPV compared to those from middle-income households in Angola (AOR: 0.56, CI 0.36–0.85, *P* < 0.01), Sierra Leone (AOR: 0.52, CI 0.31–0.87, *P* < 0.05), and Tanzania (AOR: 0.60, CI 0.42–0.87, *P* < 0.001). However, in Mozambique (AOR: 1.81, CI 1.08–3.01, *P* < 0.05) and Zambia (AOR: 1.83, CI: 1.18–2.82, *P* < 0.001), the “rich” category faced a higher risk of IPV compared to the “middle” class. Additionally, individuals in the “poor” category were more likely to experience IPV in Malawi (AOR: 2.08, CI 1.13–3.81, *P* < 0.05), Uganda (AOR: 1.58, CI 1.24–2.01, *P* < 0.001), and Zambia (AOR: 1.68, CI 1.16–2.43, *P* < 0.001). In Togo, working husbands were less likely to continue IPV during pregnancy (AOR: 0.39, CI 0.18–0.85, *P* < 0.05).

In South and Southeast Asia, the wealth index similarly influenced IPV victimization risk. In Nepal, women in the ‘rich’ category had a lower risk of experiencing IPV (AOR: 0.49, CI 0.28–0.84, P < 0.001) compared to those in the ‘middle’ class. Additionally, Working husbands had a lower likelihood of continuing IPV during pregnancy compared to the period before pregnancy (AOR: 0.34, CI 0.15–0.74, *P* < 0.001). In Latin America and the Caribbean, wealth also reduced IPV risk. For example, in Honduras, the “rich” category had a lower risk of IPV (AOR: 0.65, CI 0.50–0.84, *P* < 0.001). In North African and West Asian contexts, working husbands in Jordan had a lower likelihood of perpetrating IPV during pregnancy (AOR: 0.44, CI 0.20–0.98, *P* < 0.05) compared to non-working husbands. No significant associations were observed in Oceania for wealth or working status.

### Husband’s educational status

Education was another key determinant. In sub-Saharan Africa, spouses with primary education were at greater risk of IPV in Cameroon (AOR: 2.98, CI 1.20–7.41, *P* < 0.05) and Côte d’Ivoire (AOR: 2.58, CI 1.17–5.67, *P* < 0.05). Secondary education, however, displayed mixed results, with reduced odds of IPV in Burundi (AOR: 0.40, CI 0.22–0.74, *P* < 0.001) and increased odds in Cameroon (AOR: 3.18, CI 1.23–8.22, *P* < 0.05) and Côte d’Ivoire (AOR: 2.44, CI 1.05–5.67, *P* < 0.05). Higher education was associated with a lower likelihood of IPV in Kenya (AOR: 0.37, CI 0.17–0.85, *P* < 0.05) and Zimbabwe (AOR: 0.21, CI 0.05–0.80, *P* < 0.05). In South and Southeast Asia, secondary education was linked to decreased odds of IPV in Nepal (AOR: 0.25, CI 0.11–0.56, *P* < 0.01). In Latin America and the Caribbean, higher education reduced IPV risk in Honduras (AOR: 0.32, CI 0.14–0.72, *P* < 0.001). In North Africa and West Asia, secondary and higher education both reduced IPV risk in Egypt (AOR: 0.45, CI 0.23–0.86, *P* < 0.05; AOR: 0.18, CI 0.08–0.39, *P* < 0.001), with a similar trend in Jordan (AOR: 0.13, CI 0.02–0.93, *P* < 0.05). No significant findings were seen for Oceania.

### Spousal education disparity

Spousal education disparity also revealed varying effects on IPV risk during pregnancy. In sub-Saharan Africa, Mali showed lower odds of IPV when the spouse was more educated than the respondent (AOR: 0.39, CI 0.16–0.97, *P* < 0.05). In Latin America and the Caribbean, Honduras displayed an altered trend, with reduced IPV risk when the spouse was less educated (AOR: 0.56, CI 0.34–0.92, *P* < 0.05) and when both spouses were highly educated (AOR: 0.47, CI 0.27–0.82, *P* < 0.001). The peculiar trend was observed in Oceania, represented by Papua New Guinea. A less educated spouse increased IPV risk (AOR: 3.12, CI 1.58–6.14, *P* < 0.001), as did both spouses being highly educated (AOR: 2.58, CI 1.14–5.85, *P* < 0.05) or the spouse being more educated (AOR: 2.89, CI 1.25–6.69, *P* < 0.05). No significant findings were found for South and Southeast Asia, North Africa, or West Asia.

## Discussion

The findings of this study emphasize the significant influence of spousal educational disparities on IPV during pregnancy, shedding light on an often-overlooked factor in IPV research. While much of the existing literature on IPV has focused only on socioeconomic status, education, age, etc., this study introduces new insights by highlighting how disparities in education between partners can either exacerbate or mitigate IPV. Understanding these dynamics is crucial for developing targeted interventions to reduce IPV, particularly in low- and LMICs where gender norms and socio-economic pressures often intersect with education-related power imbalances^14^.

One of the key findings was that spousal educational disparity had varying impacts across regions. Recent studies indicated that spousal educational disparities influence IPV prevalence variably across different regions. For instance, in Sub-Saharan Africa, women with lower education levels than their spouses face a higher risk of IPV, whereas, in South Asia, women with higher education levels than their partners may experience increased IPV due to challenges to traditional gender roles^51^. In countries like Mali and Honduras, where the spouse was more educated than the respondent, the odds of IPV were significantly lower. This suggests that when the more educated spouse—often the husband—holds a higher level of education, it may foster better communication, reduce conflict, and contribute to more equitable relationship dynamics. These findings are consistent with other studies that link higher education with greater respect for women’s rights and a lower tolerance for gender-based violence^52^. However, in contrast, countries like Papua New Guinea demonstrated increased IPV risk when the spouse was more educated or when both partners were highly educated, suggesting that in certain cultural contexts, educational advancement may disrupt traditional gender roles and lead to conflict. This pattern mirrors research from other patriarchal societies, where shifts in power dynamics can create friction within relationships, resulting in heightened violence^17,53^.

Beyond educational disparities, the study also found that broader educational attainment—whether at the primary, secondary, or higher level—played a pivotal role in determining IPV risk. In sub-Saharan Africa, primary education was linked to higher IPV risk, especially in countries like Cameroon and Côte d’Ivoire. This finding aligns with global research suggesting that while primary education provides some benefits, it may not be sufficient to challenge entrenched gender norms or to empower women to assert themselves within relationships^18,54^. In contrast, secondary and higher education had a protective effect, as seen in countries like Kenya, Zimbabwe, and Honduras, where higher education among husbands and wives was associated with lower IPV risk. These results reinforce the idea that education plays a critical role in shifting gender norms and promoting more equitable relationships, thereby reducing the likelihood of violence^55^.

In addition to educational factors, the study also explored the influence of demographic and socio-economic factors on IPV during pregnancy. Age was a significant determinant in sub-Saharan Africa, where older husbands (aged 45–59) were associated with lower IPV risk, particularly in countries like Tanzania and Côte d’Ivoire. This trend reflects the broader literature on age and IPV, which suggests that older men may exhibit more mature behavior and have greater emotional stability, leading to lower levels of aggression^56–60^. However, the contrasting findings in countries like Ethiopia and Malawi, where older husbands posed a higher risk of IPV, suggest that the role of age may be mediated by other factors, such as economic hardship or cultural pressures, which can exacerbate violence.

The role of residence—urban versus rural—also yielded important insights. Literature indicated that higher IPV rates are mainly associated with rural areas. However, this study found that urban residence in countries like Angola and Malawi was linked to an increased risk of IPV^51,61^. This could be attributed to the stresses of urban living, including financial insecurity, social pressures, and overcrowding, which may strain relationships and increase the likelihood of violence^62^. These findings suggest that urbanization, while often associated with better access to education and services, can also introduce new challenges that contribute to IPV^63^.

Socio-economic status, particularly wealth, was another key factor influencing IPV risk. As expected, wealthier individuals in many countries, such as Angola and Tanzania, experienced lower IPV risk, reinforcing the idea that financial stability can buffer against the stresses that often lead to violence^64^. However, the opposite pattern observed in countries like Mozambique and Zambia, where wealth was associated with higher IPV perpetration risk, indicates that in some contexts, increased wealth may exacerbate power imbalances or create additional pressures within relationships. This finding aligns with research that suggests wealth can, in some cases, increase control and dominance within relationships, particularly in patriarchal societies^17^.

The interplay between education, socio-economic status, and IPV highlights complex dynamics influencing violence during pregnancy. This study provides innovative insights, demonstrating that spousal educational disparities significantly impact IPV risk, though their effects vary across regional contexts. In regions where education fosters gender equity, it acts as a protective factor against IPV. However, in societies with entrenched patriarchal norms, educational disparities—particularly when women attain higher education—can increase IPV risk by challenging traditional power dynamics.

These findings are critical for policymakers and public health professionals designing interventions to address intimate partner violence (IPV) during pregnancy. The results underscore the need for region-specific strategies that consider how education, socio-economic status, and demographic factors intersect to influence IPV risk. In regions where higher education contributes to reducing IPV, expanding access to education and promoting gender equity should be prioritized^65,66^. On the other hand, in areas where educational disparities between partners increase IPV, efforts must ensure that education empowers both partners and does not lead to conflict in those countries.

Future research should continue to explore the socio-cultural factors driving these patterns and evaluate the effectiveness of regionally tailored interventions. Integrating IPV prevention into education systems, promoting gender-sensitive counseling, and strengthening legal protections can contribute to more equitable and violence-free relationships. This study adds important evidence to the global understanding of IPV during pregnancy and highlights the need for nuanced approaches that address the diverse drivers of IPV across different contexts.

## Limitation and strength

While conducting this study, several limitations were noted. The use of self-reported data from the DHS surveys may introduce biases due to cultural differences in how IPV is reported and perceived. Additionally, the cross-sectional design limits the ability to establish cause-and-effect relationships. The data were collected over varying timeframes, which could affect the comparability of findings. The study also focused only on married individuals, excluding insights from non-married partners. Moreover, differences in sample sizes and population characteristics between countries may have influenced the precision and representativeness of the results. In some regions, such as Oceania, data from a single country were used to represent the entire region, limiting generalizability. Despite these limitations, this study has several strengths. It covers a large sample size across diverse geographic regions, providing valuable insights into global IPV patterns during pregnancy. The use of DHS data ensures consistency and comparability across countries, and the study highlights important regional variations that can inform policy and intervention strategies. Additionally, the focus on spousal educational disparities offers new insights into the complex dynamics of IPV, contributing to the existing body of research on gender-based violence.

## Conclusion

In conclusion, the findings confirmed that educational disparities between spouses significantly impact IPV risk, with the effect varying across different regional contexts. In some regions, higher spousal education was protective against IPV, while in others, disparities—especially when women attained higher education—were linked to increased IPV. These results emphasize the importance of considering educational disparities within couples when addressing IPV. The study also highlighted other socio-economic factors and demographic characteristics that further shape the risk of IPV, reinforcing the need for interventions tailored to specific regional and cultural contexts to mitigate IPV during pregnancy.

## Data Availability

Data is available from the Demographic and Health Surveys (DHS) program. https://dhsprogram.com.

## Abbreviations

AOR: Adjusted odds ratio
CI: Confidence interval
DHS: Demographic and health surveys
IPV: Intimate partner violence
LMIC: Lower middle-income country
PII: Personally identifiable information
PTSD: Post-traumatic stress disorder
